# Evaluation of performance and impacts of maternal and child health hospital services using Data Envelopment Analysis in Guangxi Zhuang Autonomous Region, China: a comparison study among poverty and non-poverty county level hospitals

**DOI:** 10.1186/s12939-016-0420-y

**Published:** 2016-08-23

**Authors:** Xuan Wang, Hongye Luo, Xianjin Qin, Jun Feng, Hongda Gao, Qiming Feng

**Affiliations:** School of Information and Management, Guangxi Medical University, 22 Shuang Yong Road, Qing Xiu District, Nanning, 530021 Guangxi Zhuang Autonomous Region China

**Keywords:** County-level maternal and child health hospital, Technical efficiency, Data envelopment analysis, Tobit, Influencing factors

## Abstract

**Background:**

As the core of the county-level Maternal and Child Health Hospitals (MCHH) in rural areas of China, the service efficiency affects the fairness and availability of healthcare services. This study aims to identify the determinants of hospital efficiency and explore how to improve the performance of MCHH in terms of productivity and efficiency.

**Methods:**

Data was collected from a sample of 32 county-level MCHHs of Guangxi in 2014. Firstly, we specified and measured the indicators of the inputs and outputs which represent hospital resources expended and its profiles respectively. Then we estimated the efficiency scores using Data Envelopment Analysis (DEA) for each hospital. Efficiency scores were decomposed into technical, scale and congestion components, and the potential output increases and/or input reductions were also estimated in this model, which would make relatively inefficient hospitals more efficient. In the second stage, the estimated efficiency scores are regressed against hospital external and internal environment factors using a Tobit model. We used DEAP (V2.1) and R for data analysis.

**Results:**

The average scores of technical efficiency, net technical efficiency (managerial efficiency) and scale efficiency of the hospitals were 0.875, 0.922 and 0.945, respectively. Half of the hospitals were efficient, and 9.4 % and 40.6 % were weakly efficient and inefficient, respectively. Among the low-productiveness hospitals, 61.1 % came from poor counties (Poor counties in this article are in the list of key poverty-stricken counties at the national level, published by The State Council Leading Group Office of Poverty Alleviation and Development, 2012). The total input indicated that redundant medical resources in poverty areas were significantly higher than those in non-poverty areas. The Tobit regression model showed that the technical efficiency was proportional to the total annual incomes, the number of discharge patients, and the number of outpatient and emergency visits, while it was inversely proportional to total expenditure and the actual number of open beds. Technical efficiency was not associated with number of health care workers.

**Conclusion:**

The overall operational efficiency of the county-level MCHHs in Guangxi was low and needs to be improved. Regional economic differences affect the performances of hospitals. Health administrations should adjust and optimize the resource investments for the different areas. For the hospitals in poverty areas, policy-makers should not only consider the hardware facilities investment, but also the introduction of advanced techniques and high-level medical personnel to improve their technical efficiency.

## Background

The health status of women and children reflect the level of national health, quality of civil life, and social civilization. Accelerating the development of maternal and child health care is of great importance in improving a nation’s health quality, promoting economic development and building a harmonious society. Therefore, in 2012, China's National Health and Family Planning Commission proposed a policy named the “2011–2020 China women and children development program,” which stated that funding for maternal and child health should be increased, decentralized and provided in rural and underdeveloped areas in order to promote equalization of health care services [1]. However, Differences in health service allocation in different regions is still a problem at all levels of health and family planning management. Fairness, effectiveness and accessibility of maternal and child health services is an urgent issue in rural China [2]. According to relevant literature, technical efficiency is applied to the health systems and reflects the relationship between the health utilizations (such as personnel, capital and equipment) and the health outcomes [3–8]. As the core of the maternal and child health service system, the service efficiency in MCHHs affects the supply of the maternal and child health qualities and fairness to a large extent.

In China, studies have showed that the efficiency of MCHHs are low at all levels and many researchers have said that the productivity and efficiency of MCHH needs to be improved. According to one study on 85 county-level MCHHs in Jiangsu province in 2011, 58.8 % of hospitals had a low level of efficiency [9]. In a study of 18 city-level MCHHs in Henan province of central China, the scales of compensation in most hospitals had a decline − the growth speed of output did not meet input in maternal and child health resources [10]. Based on Zhang Xiao-zhuang’s study on how district differences impact on the performance in Guangdong province, we not only analyze the overall county-level MCHH work efficiency in Guangxi province, but also take into consideration the level of socio-economic development in different areas [11]. This study aims to explore the factors affecting the efficiency of county-level MCHHs. Results from this study may help to provide strategies for the sustainable development of maternal and child health in Guangxi.

## Method

### Study setting

This study was conducted in Guangxi province, an autonomous region located in southern China with an area of 236,700 km^2^ and a 2010 population of approximately 46 million. The Han Chinese are the largest ethnic group, however over 14 million Zhuang, the largest minority ethnicity of China, live in Guangxi.

### Sampling and data collection

The questionnaire was designed according to the purpose of the survey, and mainly includes sections on input part and output. The data of the MCHH was collected from 32 counties involved in the third batch of county public hospital reforms in Guangxi, 2014. The questionnaires were issued by the Guangxi Zhuang Autonomous Region health department, and completed by the staff in county MCHHs. The questionnaires were checked for completeness and signed by the presidents of the respective hospitals. The hospitals stamped the questionnaires with the official seal and then submitted them to the County Health Bureau. The County Health Bureau audited and submitted the questionnaires to the health care reform office of the health department. The health care reform office randomly randomly selected 10 % of the questionnaires for verification.

### Data envelopment analysis (DEA) model

We set the 32 investigated county-level MCHHs as the decision-making units, where $\overline{X}$ and $\overline{Y}$ epresent the input and output data of the decision making unit (DMU) matrix. V, ω, μ and μ_0_ are the optimal value of the original planning problem, the input data, output data, and the solution of constant term, respectively. ε = 10^−6^ − 10^−4^, that is, non-Archimedes infinitely small and (1^*e*^, *n*) = (1,1,1,…,1,1,1) of the 1 × 32 matrix. When evaluating the efficiency of the j_th_ DMU, X_0_ and Y_0_ represent m different inputs, (*m* = 1, 2, 3, 4) and s different output, (*s* = 1, 2, 3) for the decision unit. Then we take the efficiency of the DMU as the target function, with all decision elements as the constraint conditions, constitute variable returns to scale model (The DEA model, which is proposed by Banker, Charnes and Cooper in 1984, is specifically designed to evaluate the effectiveness of the decision making units. Input-BCC model for short):

$$ \left\{\begin{array}{c}\hfill \max \left({\upmu}^T{Y}_0-{\upmu}_0\right)=V\hfill \\ {}\hfill {\upomega}^T\overline{X}-{\upmu}^T\overline{Y}+{\upmu}_0\begin{array}{c}\hfill \hfill \\ {}\hfill \left({1}^e,n\right)\hfill \end{array}\ge 0\hfill \\ {}\hfill {\upomega}^T{X}_0=1\hfill \\ {}\hfill \omega \ge \varepsilon \begin{array}{c}\hfill \hfill \\ {}\hfill \left({m}^e,1\right)\hfill \end{array},\mu \ge \varepsilon \begin{array}{c}\hfill \hfill \\ {}\hfill \left({s}^e,1\right)\hfill \end{array},{\upmu}_0\in {E}^1\hfill \end{array}\right. $$.

Since the model belongs to the nonlinear (fractional) programming model which is difficult to calculate, it needs to be transformed into an equivalent linear programming model, and then it turns to a dual programming model:

$$ \left\{\begin{array}{c}\hfill \min \left[\uptheta -\upvarepsilon \left(\begin{array}{c}\hfill \hfill \\ {}\hfill \left({m}^e,1\right)\hfill \end{array}{S}^{-}+\begin{array}{c}\hfill \hfill \\ {}\hfill \left({s}^e,1\right)\hfill \end{array}{S}^{+}\right)\right]=V\hfill \\ {}\hfill \overline{X}\lambda +{S}^{-}=\theta {X}_0\hfill \\ {}\hfill \overline{Y}\lambda -{S}^{+}={Y}_0\hfill \\ {}\hfill \begin{array}{c}\hfill \hfill \\ {}\hfill \left({1}^e,n\right)\hfill \end{array}\lambda =1\hfill \\ {}\hfill \lambda \ge 0,{S}^{+}\ge 0,{S}^{-}\ge 0\hfill \end{array}\right. $$

where λ is the duality programming problem solution vector and θ represents the duality programming problem relative efficiency of solution. *S*^+^ and *S*^−^. rresent the remaining variables and slack variables are introduced to solve the dual programming. When θ = 1, and all slack variables are 0, the decision unit is said to be effective. In this situation, the index values of inputs and output have reached the ideal value. According to Pareto's theory, the index is on the production frontier. If θ < 1, the unit is a weak efficiency unit, which indicates that there is excess investment and/or insufficient output [12].

### Tobit regression model

Because the relative efficiency score calculated by the DEA method ranges from 0–1, using it as the dependent variable of an ordinary least squares regression model would cause predicted values to fall outside this range. So we choose to follow the maximum likelihood estimation of the Tobit regression model to evaluate the factors that affect the efficiency of the county-level MCHH. The model is as follows [13]: Firstly, we has definition a latent variable model: *y*_i_^*^ = *β* ' *x*_*i*_ + *u*_*i*_.,here *y*_*i*_. the observable variable, then

$$ \left\{\begin{array}{c}\hfill {y}_i=0\kern0.5em  if\ {y}_i^{*}\le 0\hfill \\ {}\hfill {y}_i={y}_i^{*}\kern0.75em  if\ 0<{y}_i^{*}<1\hfill \\ {}\hfill {y}_i=1\kern0.5em  if\ {y}_i^{*}\ge 1\hfill \end{array}\right. $$.

### Instruments

In order to accurately measure the relative productivity and efficiency of public hospitals in Guangxi, it is crucial to select a group of appropriate input and output variables. Since the DEA model is actually a linear system based on an optimization model, the solution of the DEA model is in fact the two suitable weight vector (ω, μ) [12]. Therefore, in the DEA model, the previous studies have chosen the absolute index. Based on a review of related literature, the DEA model in this study selected total expenditure, the total number of hospital doctors, nurses and actual open beds as input indicators, and the total income, the number of patient discharges, the number of outpatients and emergency visits as output indicators (refer to the standard explanation, Table 1) [14–16]. In the second step, the 7 indicators above were selected as independent variables into the Tobit regression model to determine the factors associated with the efficiency of county-level MCHHs in Guangxi.

**Table 1:** Input and output index in DEA model

Category	Variable	Definition
Input	Total expenditure	Capital consumption and defray in the process of service provision and other activities, including healthcare expenditure, drug and medicine expenditure.
	Number of doctors	Registered doctors at the end of year, excluding retirees and temporary staff
	Number of nurses	Registered nurses at the end of year, excluding retirees and temporary staff
	Number of open beds	The number of available bed days divided by the number of days in a year
Output	Total revenue	Revenue gained from service provision and other activities, including healthcare revenue, drug and medicine sales, financial subsidies.
	Number of outpatients and emergency visits	The number of patients coming for outpatient and emergency diagnostic services
	Number of discharged patients	The number of discharged patients after hospitalization for various reasons

## Results

### Basic information of the input and output indicators

From Table 2 it can be seen that all of the evaluation indices of the MCHH in poor counties were much lower than the non-poverty counties. The difference among number of outpatients and emergency visits was significance (*P* < 0.05). And other indicators, may be subject to the reasons for the sample content, the difference was not statistically significant.

**Table 2:** Comparison of sample indicators (median)

	Poor counties	Non-poor counties	*p*-value
Total revenue (10 thousand, RMB)	1,660	2,421	0.07
Number of discharged patients	2,759	4,026	0.14
Number of outpatients and emergency visits	38,343	68,049	0.02
Total expenditure (10 thousand, RMB)	1450	2128	0.12
Number of doctors	21.2	26.7	0.14
Number of nurses	36.7	46	0.32
Number of open beds	64.4	68	0.82

### Results of the DEA model

#### Operational efficiency of the 32 county-level MCHHs

As shown in Table 3, 16 hospitals had efficiency scores of 1.0 for comprehensive, technical and scale efficiencies, and the scale of compensation was constant. The slack variable was 0, that is, allocation of health resources in the 16 hospitals were relatively effective (including 7 poor counties and 9 non-poor counties). Three hospitals had technical efficiency scores of 1.0, scale efficiency scores of less than 1.0, a slack variable of 0, and a reduced scale of compensation. In other words, the efficiency of health resource allocation in these 3 hospitals is weakly efficient (including 2 poor counties and 1 non-poor county). The health resource allocation efficiency of 13 hospitals were relatively low, and their scale of compensation increased or decreased, their technical and scale efficiency scored less than 1.0, and not all the slack variables were 0 (including 9 poor counties and 4 non-poor counties).

**Table 3:** Efficiency score and returns-to-scale characteristics of each DMU

DMU	Crste Score	Vrste Score	Scale Efficiency Score	Type of scale inefficiency	DMU	Crste Score	Vrste Score	Scale Efficiency Score	Type of scale inefficiency
X1^a^	1.000	1.000	1.000	-	X17^a^	1.000	1.000	1.000	-
X2^a^	0.945	0.966	0.978	drs	X18^a^	1.000	1.000	1.000	-
X3^a^	0.592	0.780	0.759	irs	X19^a^	0.691	0.702	0.985	drs
X4^a^	1.000	1.000	1.000	-	X20	0.602	0.625	0.963	drs
X5^a^	0.906	0.954	0.949	irs	X21	1.000	1.000	1.000	-
X6^a^	0.675	0.715	0.944	irs	X22	0.748	0.754	0.992	irs
X7^a^	0.918	1.000	0.918	irs	X23^a^	1.000	1.000	1.000	-
X8	1.000	1.000	1.000	-	X24	1.000	1.000	1.000	-
X9^a^	0.880	1.000	0.880	irs	X25	1.000	1.000	1.000	-
X10	1.000	1.000	1.000	-	X26^a^	1.000	1.000	1.000	-
X11	1.000	1.000	1.000	-	X27	0.965	0.979	0.985	drs
X12^a^	1.000	1.000	1.000	-	X28^*^	0.799	0.811	0.986	drs
X13	1.000	1.000	1.000	-	X29	0.853	1.000	0.853	irs
X14^a^	0.733	0.832	0.880	drs	X30	1.000	1.000	1.000	-
X15^a^	0.521	0.879	0.593	irs	X31	1.000	1.000	1.000	-
X16^a^	0.612	0.682	0.898	irs	X32	0.560	0.815	0.688	irs
mean	0.875	0.922	0.945						

^a^ Poor counties constant return to scale; irs: increasing return to scale; drs: decreasing return to scale

#### Slack variable analysis

In the BCC model, we calculated both the technical efficiency of each DMU and the projection value of the input and output indicators of the inefficient hospitals. By determining the differences between the actual value and the projection value, we obtained the relaxation of the index which can provide the quantitative basis for the improvement of the production status of the decision making unit.

##### Analysis of slack variable of the MCHH in poor counties

From Table 4, if these 9 inefficient hospitals in poor counties aim at achieving the Pareto optimality, they have to reduce the total expenditure by an average of 0.854 million yuan, reduce the number of doctors by 7.6, nurses by 11.3 and open beds by 11.5. They also need to increase the average total income by 17,000 yuan, increase the number of discharged patients by 723 and increase outpatient and emergency visits by 15,455.6.

**Table 4:** Average redundancy of the MCHHs in poor and non-poor counties

	Poor counties	Non-poor counties
Total revenue (10 thousand, RMB)	1.7	6.8
Number of discharged patients	723	149.9
Number of outpatient and emergency visits	15455.6	29057.6
Total expenditure (10 thousand, RMB)	85.4	37.5
Number of doctors	7.6	1.8
Number of nurses	11.3	4.4
Actual number of open beds	11.5	9.2

##### Analysis of slack variable of the MCHHs in non-poverty counties

From Table 4, if these 4 inefficient hospials in non-poor counties aim at achieving the Pareto optimality, they have to reduce the average total expenditure by 0.375 million yuan, reduce the number of doctors by 1.8, nurses by 4.4 and open beds by 9.2. They also need to increase the average total income by 68,000 yuan, increase the number of discharged patients by 149.9 and outpatient and emergency visits by 29,057.6.

### Tobit regression model

Taking the overall technical efficiency score value as the dependent variable and the DEA model as the independent variable, we used Tobit regression model to analyze the main factors affecting comprehensive efficiency. From Table 5, when the test levelα = 0.05, the overall technical efficiency of the county-level MCHHs in Guangxi was proportional to the GDP per capita and the number of health professionals, while it was inversely proportional to the number of open beds. County population and total expenditure were not associated with overall technical efficiency.

**Table 5:** Estimation results for Tobit regression model

crste score variable	Coeficient	Standar d Error	*P* value	95 % Confidence interval	
County population	−0.001654	0.001156	0.164	(−0.004026	0.000719)
GDP per capita	0.057936	0.028070	0.049	(0.000340	0.115531)
Number of open beds	−0.002823	0.001023	0.010	(−0.004922	−0.000724)
Number of health professionals	0.004665	0.001176	0.000	(0.002252	0.007078)
Total expenditure	0.000011	0.000030	0.722	(−0.000051	0.000072)
Constant	0.551086	0.074430	0.000	(0.398369	0.703803)

## Conclusion

Half of the maternal and child health hospitals are on the frontier, and their health utilizations had achieved a relatively optimal output value (38.9 % for hospitals in poor counties and 64.3 % for those in non-poor counties,). More than 40 % of hospitals were found to be inefficient and about 10 % were weakly efficient.with increasing scales of compensation. This shows that the sizes of the existing health resources in the weakly efficient hospitals were relatively smaller than others and the growth rate of output were higher than that in investment. In other words, at their current sizes, they were not at the optimal combination of production factors, so more investment should be put into them. For the inefficient hospitals, health resource allocation is relatively ineffective, that is, at the current size, the investment resources are not fully utilized, and they cannot achieve the best scale of the state. From the overall comprehensive efficiency, only half of the county-level hospitals were efficient, which represents a low utilization efficiency of maternal and child health services in counties of Guangxi. These results are consistent with Li Lianfeng’s study, which showed that in 2013, 71.4 % of the urban hospitals in Guangxi were relatively ineffective [17]. Increased health care resources to these hospitals should be a priority. Because the 3 hospitals in this article are increasing returns to scale. And the hospitals categorized as relatively ineffective must ameliorate their management strategies and improve their medical services.

According to slack variable analysis of input indexes, the input redundancy of maternal and child health hospitals in poor counties is much higher than that in non-poor counties. In this study, 61.1 % of hospitals in poor counties are in a weakly effective or inefficient state, so the input of health resources has not been fully utilized. Although hardware facilities have been improved since the medical reform in 2009, hospitals often encounter obstacles, such as shortages of medical professionals, which can reduce their efficiency. This suggests that when the health administrators make regional health plans, they need to consider the input redundancy of MCHHs in impoverished counties, involve hardware investments and medical technology and introduce high-level personnel to improve their health services. From the analysis of the slack variables of output, there would be greater improvement through increasing the number of inpatients in the MCHHs in poor counties than those in non-poor counties. On account of the connection between medical utilization and socio-economic development, people are more likely to fail to get access to the health care services in poor counties due to the mountain environment and inconvenient transportation, and it will therefore restrict improvement of their output.

There have been many studies exploring factors affecting the efficiency of general hospitals, but few have explored the mechanisms of these factors. A study exploring the internal factors influencing the efficiency of MCHHs in Guangdong Province found that the quality of health staff was the most important factor for improving the efficiency of hospitals [18]. Environmental factors were also found to be associated with efficiency, for example new health care reform policies, population size, outpatient expenses per capita, and proportion of prescribed drug had a significant negative effect, while net income per capita of rural residents, hospitalization expenses per capita, and asset turnover rate, had a significant positive effect on efficiency [19]. The results of our study are somewhat similar to the research we mentioned above. Internal and external environmental factors of hospital indicates the reasons why the county-level MCHH’s failed to attract and retain patients; mainly from shortages of basic facilities, funding, equipment, qualified personnel, technology and hospital incentive mechanisms. So all MCHH levels should continue to strengthen their infrastructure through the introduction of qualified external personnel and the training of internal personnel, increase the proportion of highly-educated health workers, enhance the capacity of health services, and improve service quality. Our study did not find some factors mentioned in other studies such as cure rate, return on assets, and drug income, probably because the different functions of the MCHH in different levels will lead to a different construction of the DEA model. In addition, the relative efficiency values cannot be compared directly. In conclusion, the influencing factors can affect the county-level MCHHs technical efficiency and need to be further studied.

## Abbreviations

MCHH, Maternal and Child Health Hospital; DEA, data envelopment analysis; DMU, decision-making unit
